# The Netherlands Cohort Study – Meat Investigation Cohort; a population-based cohort over-represented with vegetarians, pescetarians and low meat consumers

**DOI:** 10.1186/1475-2891-12-156

**Published:** 2013-11-29

**Authors:** Anne MJ Gilsing, Matty P Weijenberg, R Alexandra Goldbohm, Pieter C Dagnelie, Piet A van den Brandt, Leo J Schouten

**Affiliations:** 1 Department of Epidemiology, GROW-School for Oncology and Developmental Biology, Maastricht University, PO Box 616, Maastricht 6216, MD, The Netherlands; 2 TNO, Leiden, The Netherlands; 3 Department of Epidemiology, CAPHRI School of Public Health and Primary Care, Maastricht University, Maastricht, The Netherlands

**Keywords:** Vegetarian, Low meat diet, Cohort, Self-report, FFQ

## Abstract

**Background:**

Vegetarian diets have been associated with lower risk of chronic disease, but little is known about the health effects of low meat diets and the reliability of self-reported vegetarian status. We aimed to establish an analytical cohort over-represented with vegetarians, pescetarians and 1 day/week meat consumers, and to describe their lifestyle and dietary characteristics. In addition, we were able to compare self-reported vegetarians with vegetarians whose status has been confirmed by their response on the extensive food frequency questionnaire (FFQ).

**Study methods:**

Embedded within the Netherlands Cohort Study (n = 120,852; including 1150 self-reported vegetarians), the NLCS-Meat Investigation Cohort (NLCS-MIC) was defined by combining all FFQ-confirmed-vegetarians (n = 702), pescetarians (n = 394), and 1 day/week meat consumers (n = 1,396) from the total cohort with a random sample of 2–5 days/week- and 6–7 days/week meat consumers (n = 2,965 and 5,648, respectively).

**Results:**

Vegetarians, pescetarians, and 1 day/week meat consumers had more favorable dietary intakes (e.g. higher fiber/vegetables) and lifestyle characteristics (e.g. lower smoking rates) compared to regular meat consumers in both sexes. Vegetarians adhered to their diet longer than pescetarians and 1 day/week meat consumers. 75% of vegetarians with a prevalent cancer at baseline had changed to this diet after diagnosis. 50% of self-reported vegetarians reported meat or fish consumption on the FFQ. Although the misclassification that occurred in terms of diet and lifestyle when merely relying on self-reporting was relatively small, the impact on associations with disease risk remains to be studied.

**Conclusion:**

We established an analytical cohort over-represented with persons at the lower end of the meat consumption spectrum which should facilitate prospective studies of major cancers and causes of death using ≥20.3 years of follow-up.

## Introduction

The popularity of (semi-)vegetarianism is rapidly increasing in the western world as a result of nutritional, ethical, and more recently, environmental concerns [1]. At the same time, the world-wide per capita meat consumption is also expected to increase considerably over the next decades [2], especially in developing countries [3,4].

Although this transition has been accompanied by an increasing scientific interest in the health effects of vegetarian diets, only a small number of large prospective cohort studies specifically set out to study these by including a large proportion of vegetarians [5-10]. Thus far, these studies provided convincing evidence that vegetarians have a lower risk of coronary heart disease [11], a possibly lower risk of diabetes [12], metabolic syndrome [13], and some types of cancer [14], and a greater life expectancy [11] compared to meat eaters. These observed health benefits may largely be explained by a more favorable distribution of chronic disease risk factors such as overweight [15,16], blood lipids [17], and blood pressure [18] among the vegetarians.

Despite these scientific advancements, no universally accepted definition for the term vegetarian exists and there is substantial inconsistency in how people self-identify being vegetarian [19,20]. This ambiguity may hinder the comparability of previous studies. Only a few studies identified vegetarians based on extensive dietary data collection techniques [5,6] whereas others reported to have classified vegetarians by means of several broader questions relating to the overall consumption of animal products [7-9], or to have used self-reported vegetarianism [10] as inclusion criterion. Even though it has been shown that self-reported vegetarians are generally health conscious, several studies suggest that this group still includes a considerable number of occasional meat consumers [19,20] which may have biased epidemiological study results. Therefore, the usefulness and reliability of self-reported vegetarian status in observational studies needs to be further evaluated. Moreover, further work is needed in studies with a large number of vegetarians and low meat eaters to address whether complete abstinence of meat might have an additional beneficial health effect over low meat consumption, or vice versa.

Therefore, we have defined an analytical cohort specifically designed to study the health effects of vegetarian and low meat diets focusing on cancer incidence and mortality, embedded within a large ongoing prospective cohort that used an extensive FFQ to assess dietary intake. In the current paper we present the description of the baseline characteristics of this analytical cohort that is overrepresented with low and no meat consumers. We performed a cross-sectional comparison of lifestyle factors, dietary intake, and prevalent cancer status across the various meat consumption categories. In addition, we performed a cross comparison of self-reported vegetarians and vegetarians whose status has been confirmed by their response on the FFQ.

## Subjects and methods

The prospective Netherlands Cohort Study on diet and cancer (NLCS) was initiated in 1986 with the purpose of investigating the association between diet and cancer. The details of the study have been described elsewhere [21]. The study population originated largely from 204 municipal population registries throughout the country. In addition, vegetarians were overrepresented by intentionally contacting them through health food shops and magazines. This method of recruitment resulted in the inclusion of 685 vegetarians and low meat consumers. A total of 58,279 men and 62,573 women between the ages of 55 and 69 years completed a mailed, self-administered questionnaire on dietary habits and other risk factors for cancer, at baseline. The NLCS has been approved by the institutional review boards of the TNO Quality of Life Research Institute (Zeist, the Netherlands) and Maastricht University (Maastricht, the Netherlands). Because the NLCS traditionally uses a case-cohort approach for reasons of efficiency in questionnaire processing, the baseline questionnaires were only entered for all failures (i.e. incident cancer cases) and a random subcohort of 5,000 individuals that was chosen immediately after baseline. The first page of the questionnaire was entered and processed for all 120,852 participants.

### NLCS-MIC

We defined an analytical cohort, embedded within the NLCS, that is specifically designed to study the health effects of vegetarian and low meat diets; the NLCS ‘Meat Investigation Cohort’ (NLCS-MIC). The NLCS-MIC was created by expanding the random subcohort of the NLCS from 5,000 to 10,000 individuals (to increase power) and combining these with all the (self-reported) vegetarians and individuals who consumed meat 1 day/week from the NLCS. The latter groups were identified based on two items on the first page of the questionnaire relating to specific dietary regimens: “how many days on average per week do you eat meat?”, and “Do you have any special eating habits?” The remainder of the questionnaire, including the 150 item semi-quantitative food frequency questionnaire (FFQ), was also entered and processed for this group of vegetarians and low meat consumers. The detailed dietary data was used to further categorize NLCS-MIC into five meat consumption categories (non-meat consumers based on FFQ (divided into vegetarians and pescetarians), 1 day/week- , 2–5 days/week-, and 6–7 days/week meat consumers), as described below.

### Meat consumption questions

The NLCS-FFQ that was used to categorize NLCS-MIC, contained 14 items on the consumption of meat with the hot meal (mainly fresh meat, including chicken), 5 items on the consumption of meat products used as sandwich fillings, and 3 items on fish consumption. A validation study conducted in a subgroup of the cohort two years after the baseline measurement indicated that the Spearman correlation coefficients for meat, meat products and fish, as assessed by the questionnaire, and those estimated from the 9-day dietary record were 0.46, 0.54 and 0.53 respectively. The number of vegetarians and individuals consuming meat1 day/week was too low in this validation sample to assess the above correlations in these selected groups [22]. In addition, the questionnaire also assessed the time since the start of any special eating habits and weekly frequency of meat consumption (for 0–1 day/week meat consumers), in years prior to baseline (1986).

### Classification of NLCS-MIC

NLCS-MIC was classified based on the FFQ as depicted in Additional file 1: Figure S1.

#### Classification of non-vegetarians

Individuals who reported to eat ≥1 type of meat with the hot meal (14 items on FFQ) were categorized based on their self-reported weekly meat consumption frequency as indicated in the question (“how many days on average per week do you eat meat?”) into 1 day/week, 2–5 days/week, and 6–7 days/week meat consumers.

#### Classification of “FFQ confirmed vegetarians and pescetarians”

We defined ‘FFQ confirmed vegetarians’ (later referred to as ‘confirmed-vegetarians’) as individuals who reported to consume a diet void of meat and fish on the extensive FFQ (including vegans, lactoovo-, lacto-, and ovo-vegetarians). In order to be classified as non-meat consumers individuals had to fulfill the following three criteria simultaneously: 1) not eating any meat items for the hot meal (14 items FFQ), 2) abstaining from meat as sandwich fillings (5 items FFQ), and 3) consuming meat for 0 days/week as indicated in the question (“how many days on average per week do you eat meat?”). In addition, individuals adhering to criteria 1 and 2 with a missing on criterion 3 who indicated to adhere to a non-meat dietary regimen (i.e. vegetarian/vegan), were also considered to be non-meat consumers. All non-meat consumers were subsequently categorized to be either vegetarian or pescetarian (fish eater) based on the 3 items relating to fish consumption.

#### Classification of self-reported vegetarians

Because we wanted to examine the reliability of self-reported vegetarianism as compared to a vegetarian status confirmed by the FFQ, we additionally identified all self-reported vegetarians based on the question: “Do you have any special eating habits?”. Individuals who reported to adhere to a vegetarian, vegan, or Seventh Day Adventist diet were classified as self-reported vegetarians.

Due to the case-cohort design of the original NLCS cohort, NLCS-MIC consists of a random sample of 10,000 individuals (with random meat consumption), and all the vegetarians and low meat consumers from the total cohort. As a result, all self-reported vegetarians who reported eating meat >1 day/week and who were not part of the randomly selected subcohort (n = 119), were only included in analyses when comparing self-reported vegetarians to the complementary group of non-(self-reported) vegetarians, and not for all other contrasts.

### Incomplete and inconsistent dietary data

Within the NLCS, participants with incomplete and inconsistent dietary data are excluded from analyses [22]. These exclusion criteria were based on the number of blank and marked items, but not designed for use in non-meat eating populations. As vegetarians left meat-related items blank, they automatically meet these exclusion criteria more easily than those who consumed meat in their diet. Therefore, new cut-off values for non-meat consumers, using the same underlying principles as for the total NLCS cohort, were established. For all non-meat consumers, the cut-off criteria for incomplete questionnaires were set at >90 blank- and <22 consumed items, whereas meat eaters were scored according to the original NLCS values (>60 items and <35, respectively). Out of the 135 non-meat eaters with incomplete questionnaires, 39 were no longer considered to be incomplete after implementing these new scores. The proportion of incomplete questionnaires was 6% among the random sample of 10,000 and 7% among the non-meat eaters. Finally, NLCS-MIC consists of 11,867 cohort members, including 1,227 non meat consumers (785 vegetarians and 442 pescetarians) and 1,499 participants who consume meat for only 1 day/week.

### Statistical analyses

Dietary and lifestyle characteristics were described for the five meat consumption categories (confirmed-vegetarians and -pescetarians, and individuals consuming meat 1 day/week, 2–5 days/week, and 6–7 days/week). We also examined differences in dietary and lifestyle characteristics between the classifications of vegetarian status, i.e. between self-reported and FFQ confirmed vegetarians.

Because of the relatively high number of cancer survivors among the vegetarians, pescetarians and individuals consuming meat 1 day/week, all prevalent cancer cases at baseline were excluded from the main analyses and described as a separate group. All analyses were conducted for men and women separately. All nutrient intake variables were adjusted for energy intake by the residual method [23]. Differences between the five meat consumption categories were assessed using chi-square tests for categorical variables, and one-way analyses of variance (ANOVA) for continuous variables.

Due to a skewed distribution of some dietary intake variables we transformed these variables prior to performing analyses of variance, using the log normal transformation. Non-parametric tests were also applied to not normally distributed data (untransformed) (Kruskal-Wallis test), and because these results were comparable to those from ANOVA, the latter was applied to all items for consistency.

All analyses were performed with STATA Statistical Software (Intercooled version 11; StataCorp LP, College Station, TX). All tests were 2-tailed, and differences were regarded as statistically significant at *P* <0.05.

## Results

### The NLCS-MIC

After excluding prevalent cancer cases at baseline, the NLCS-MIC includes 1,150 self-reported vegetarians, of whom 50% were also classified as vegetarian according to the information on the FFQ (n = 574; 194 men and 380 women); the remaining 50% of self-reported vegetarians reported to eat meat and/or fish on the FFQ. Moreover, an additional 23 males and 104 females were classified as vegetarians according to the FFQ but did not define themselves as being vegetarian, resulting in a total of 702 confirmed-vegetarians (Table 1). In addition, the NLCS-MIC also includes 394 pescetarians, 1,396 individuals consuming meat only 1 day/week, 2,965 individuals eating meat 2–5 days/week and 5,649 individuals who consume meat six or seven days/week (see also Additional file 1: Figure S1).

**Table 1:** **Self-reported vegetarian status at baseline of members of the NLCS-Meat Investigation Cohort (NLCS-MIC) by meat consumption status**^**a**^

		**All confirmed vegetarians and low meat consumers from the total NLCS cohort**			**Random sample of meat consumers from the total NLCS cohort**^**b**^			
			
		**Vegetarians**	**Pescetarians**	**1 day/wk**	**2–5 day/wk**	**6–7 day/wk**	**Remainder**^**c**^	**Total**
Men	Self-reported vegetarians	194 (47%)^d^	96 (23%)	71(17%)	5 (1%)	1 (0.3%)	49 (12%)	416
	Non self-reported vegetarians	23 (0.5%)	56 (1%)	411 (9%)	1321 (28%)	2972 (62%)	N/A	
	Total	217	152	482	1326	2973	49	
Women	Self-reported vegetarians	380 (52%)	151 (21%)	132 (18%)	7 (1%)	4 (0.6%)	60 (8%)	734
	Non self-reported vegetarians	105 (2%)	91 (2%)	782 (15%)	1632 (31%)	2672 (51%)	N/A	
	Total	485	242	914	1639	2676	60	
Total		702	394	1396	2965	5649	109	

^a^Excluding prevalent cancer cases at baseline.

^b^Sampling rate is 8.3%.

^c^Self-reported vegetarians eating meat >1 day/wk and outside random sample (see also step 6 Additional file 1: Figure S1).

^d^Percentage of self-reported vegetarians, all such values.

### Comparison of the five diet groups based on the FFQ

Data on lifestyle and reproductive characteristics by meat consumption category are presented in Table 2. No difference in age was observed between the diet groups. Vegetarians and pescetarians were more often female. Mean BMI was lowest amongst pescetarians and vegetarians and increased with increasing meat intake. In both men and women, those who reported not to consume meat had, on average, a higher level of education, and were least likely to be married or current smokers. Age at menarche was lowest in the vegetarian women. On average, vegetarians had the lowest number of children (data only available for women). Among men, those who reported not to consume meat had more frequent bowel movements compared to meat eaters (P < 0.001), while fish eaters had the lowest rates of constipation (P < 0.001) (data only available for men) (Table 3). Among women, vegetarians and pescetarians more often considered their own health status to be excellent (20%) compared to all the meat eating groups (~14%). Detailed data on intake (in g/day) of major food groups, selected foods and macronutrients in the five meat consumption groups are shown in Table 4; intake of all items was significantly different across the groups (P < 0.001) in both men and women. In short, total energy intake was lowest in those who reported to consume meat 1 day/week, followed by vegetarians, pescetarians, and highest in individuals consuming meat more than 6 days/week. The vegetarians and pescetarians consumed the largest amounts of fruits, vegetables, pulses, grains, nuts and seeds, soy products, dairy and cheese; consumption of these products was lowest among 2–5 and 6–7 days/week meat eaters, while individuals who reported to consume meat 1 day/week had intakes at in-between levels. Mean fish intake decreased from the low to high meat intake groups and was highest amongst the pescetarians. Individuals in the highest meat eating group (6–7 days/week) consumed alcohol at levels almost three times as high as the vegetarians. Data on daily intake of vitamins and minerals in the five meat consumption groups are shown in Table 5. Intake of all these micronutrients was significantly different across the diet groups (P < 0.001). Intake of vitamins A, B2, C, E, carotene, calcium, folate, phosphorus, and dietary fiber was highest among the vegetarians and decreased with increasing intake of meat. The opposite pattern was observed for intake of vitamin B6 and cholesterol in the diet, which was highest among those consuming meat 6–7 days/week. The amount of salt added to the meal during cooking increased considerably with increasing meat intake; individuals consuming meat 6–7 days/week added nearly three times the amount of salt compared to vegetarians.

**Table 2:** **Lifestyle and reproductive characteristics of members of the NLCS-Meat Investigation Cohort by meat consumption status**^**a**^

	**Men**							**Women**						
	**All confirmed vegetarians, pescetarians and low meat consumers from the total cohort**			**Random sample of meat consumers from the total cohort**			**Self-reported vegetarians**	**All confirmed vegetarians, pescetarians and low meat consumers from the total cohort**			**Random sample of meat consumers from the total cohort**			**Self-reported vegetarians**
	**Vegetarian**	**Pescetarian**	**1 day/wk**	**2-5 day/wk**	**6-7 day/wk**			**Vegetarian**	**Pescetarian**	**1 day/wk**	**2-5 day/wk**	**6-7 day/wk**		
	**n = 217**	**n = 152**	**n = 482**	**n = 1326**	**n = 2973**	***P***^***b***^	**n = 416**	**n = 485**	**n = 242**	**n = 914**	**n = 1639**	**n = 2676**	***P***^***b***^	**n = 734**
Age (y)	60.6 ± 4.0^c^	60.8 ± 3.7	61.3 ± 4.3	62.6 ± 4.2	62.3 ± 4.2	0.21	61.0 ± 4.2	61.8 ± 1.1	60.7 ± 4.3	61.5 ± 4.2	61.5 ± 4.3	61.4 ± 4.3	0.83	61.3 ± 4.2
BMI (kg/m2)	23.4 ± 2.6	23.4 ± 2.5	24.2 ± 2.7	24.8 ± 2.5	25.1 ± 2.6	<0.001	23.4 ± 2.5	23.1 ± 3.1	22.7 ± 3.0	23.9 ± 3.6	24.9 ± 3.5	25.2 ± 3.5	<0.001	23.1 ± 3.1
Physical activity, non-occupational (min/day)	72.0 ± 45	76.1 ± 47	80.2 ± 65	79.0 ± 62	79.1 ± 69	0.57	76.6 ± 51	67.8 ± 42	67.1 ± 50	66.2 ± 51	66.4 ± 54	62.7 ± 51	0.067	68.9 ± 48
Current smokers (%)	16%	11%	23%	35%	37%	<0.001	13%	9%	15%	19%	24%	19%	<0.001	10%
Level of Education														
Lower vocational	21%	24%	33%	45%	45%	<0.001	23%	29%	34%	43%	53%	59%	<0.001	27%
Second and medium vocational	32%	32%	34%	35%	35%		33%	48%	41%	42%	33%	33%		48%
University and higher vocational	47%	43%	33%	20%	19%		43%	23%	24%	16%	9%	7%		24%
Marital status														
Married (%)	77%	87%	79%	89%	90%	<0.001	80%	55%	61%	53%	65%	74%	<0.001	55%
Reproductive factors (women only)														
Age at menarche (y)								13.3 ± 1.5	13.6 ± 1.9	13.5 ± 1.8	13.6 ± 1.7	13.7 ± 1.8	<0.001	13.4 ± 1.7
Age at menopause (y)								48.9 ± 4.1	48.9 ± 4.6	48.6 ± 4.6	48.7 ± 4.4	48.7 ± 4.5	0.75	48.8 ± 4.4
Use of oral contraceptives (% yes)								22%	33%	27%	25%	25%	0.017	29%
Use of post-menopausal hormones (% ever)								11%	14%	14%	14%	13%	0.19	13%
Age at first birth (y)								27.1 ± 4.1	27.4 ± 4.2	26.7 ± 4.5	26.8 ± 4.2	27.0 ± 4.3	0.10	27.2 ± 4.1
Parity (number of children)								2.3 ± 2.1	2.5 ± 2.0	2.5 ± 2.2	2.8 ± 2.2	2.8 ± 2.2	<0.001	2.3 ± 2.1

^a^Excluding prevalent cancer cases at baseline.

^b^P values are derived from one way anova for continuous variables and from a chi2 test for categorical variables comparing the five meat consumption groups (vegetarian, pescetarian, 1 day/wk-, 2–5 day/wk-, and 6–7 day/wk meat consumers).

^c^Mean ± SD, all such values.

**Table 3:** Hours of sleep, perceived general health, family history of cancer, cancer screening participation, and bowel movement of members of the NLCS-Meat Investigation Cohort by meat consumption status

	**Men**							**Women**						
	**All confirmed vegetarians, pescetarians and low meat consumers from the total cohort**			**Random sample of meat consumers from the total cohort**			**Self-reported vegetarians**	**All confirmed vegetarians, pescetarians and low meat consumers from the total cohort**			**Random sample of meat consumers from the total cohort**			**Self-reported vegetarians**
	**Vegetarian**	**Pescetarian**	**1 day/wk**	**2–5 day/wk**	**6–7 day/wk**			**Vegetarian**	**Pescetarian**	**1 day/wk**	**2–5 day/wk**	**6–7 day/wk**		
	**n = 217**	**n = 152**	**n = 482**	**n = 1326**	**n = 2973**	***P***^***b***^	**n = 416**	**n = 485**	**n = 242**	**n = 914**	**n = 1639**	**n = 2676**	***P***^***b***^	**n = 734**
Hours of sleep per day (h)	7.8 ± 1.1^c^	7.7 ± 0.9	7.8 ± 1.1	7.8 ± 1.1	7.8 ± 1.1	0.34	7.8 ± 1.1	7.6 ± 1.1	7.6 ± 1.1	7.5 ± 1.3	7.6 ± 1.2	7.6 ± 1.1	0.28	7.6 ± 1.1
Perceived general health (%)														
Excellent	25%	22%	19%	18%	18%	0.12	24%	20%	20%	14%	13%	13%	<0.001	20%
Reasonable/good	69%	72%	71%	75%	74%		72%	70%	67%	72%	77%	79%		70%
Poor/bad	6%	7%	10%	7%	8%		4%	10%	13%	14%	9%	7%		11%
Family history of cancer at baseline (first degree) (% yes)	40%	47%	45%	43%	46%	0.57	42%	47%	43%	50%	46%	48%	0.20	48%
Cancer screening (women only)														
Cervical Smear (% yes)								73%	73%	74%	76%	73%	0.50	74%
Breast cancer screening (% yes)								23%	25%	29%	32%	28%	0.031	26%
Bowel movement (men only)														
>2 times per day	7%	5%	5%	3%	3%	<0.001	6%							
1–2 times per day	43%	46%	35%	34%	35%		44%							
Once a day	48%	45%	54%	57%	56%		47%							
Every two days or less	2%	3%	6%	7%	6%		3%							
Constipation frequency (men only)														
Never	55%	62%	46%	53%	56%	0.008	53%							
Seldom	36%	27%	40%	34%	33%		36%							
Sometimes or more often	9%	11%	13%	13%	11%		12%							

^a^Excluding prevalent cancer cases at baseline.

^b^P values are derived from one way anova for continuous variables and from a chi2 test for categorical variables comparing the five meat consumption groups (vegetarian, pescetarian, 1 day/wk-, 2–5 day/wk-, and 6–7 day/wk meat consumers).

^c^Mean ± SD, all such values.

**Table 4:** **Mean daily intake of selected food groups, foods , and nutrients of members of the NLCS-Meat Investigation Cohort by meat consumption status**^**a**^

	**Men**							**Women**						
	**All confirmed vegetarians, pescetarians and low meat consumers from the total cohort**			**Random sample of meat consumers from the total cohort**			**Self-reported vegetarians**	**All confirmed vegetarians, pescetarians and low meat consumers from the total cohort**			**Random sample of meat consumers from the total cohort**			**Self reported vegetarians**
	**Vegetarian**	**Pescetarian**	**1 day/wk**	**2–5 day/wk**	**6–7 day/wk**			**Vegetarian**	**Pescetarian**	**1 day/wk**	**2–5 day/wk**	**6–7 day/wk**		
	**n = 217**	**n = 152**	**n = 482**	**n = 1326**	**n = 2973**	***P***^***b***^	**n = 416**	**n = 485**	**n = 242**	**n = 914**	**n = 1639**	**n = 2676**	***P***^***b***^	**n = 734**
Energy (kcal)	1985 ± 515	2082 ± 540	1926 ± 548	2042 ± 494	2215 ± 501	<0.001	2018 ± 521	1575 ± 413	1581 ± 427	1571 ± 464	1646 ± 393	1723 ± 395	<0.001	1625 ± 420
Food groups (g/day)														
Vegetables	235 ± 100	248 ± 107	207 ± 105	189 ± 83	193 ± 84	<0.001	245 ± 102	225 ± 101	250 ± 103	211 ± 111	195 ± 82	193 ± 81	<0.001	246 ± 101
Fruit	227 ± 156	238 ± 188	177 ± 144	162 ± 120	149 ± 109	<0.001	229 ± 159	252 ± 157	235 ± 145	214 ± 143	193 ± 120	192 ± 119	<0.001	246 ± 143
Pulses	28.0 ± 43.7	27.0 ± 34.2	16.7 ± 23.7	10.1 ± 14.3	8.9 ± 12.2	<0.001	25.8 ± 38.0	17.0 ± 26.9	17.8 ± 27.2	12.3 ± 20.2	7.4 ± 9.4	6.3 ± 9.1	<0.001	16.9 ± 21.8
Grains	18.0 ± 25.4	19.0 ± 26.8	8.4 ± 14.8	2.4 ± 7.2	1.3 ± 4.9	<0.001	16.1 ± 23.1	16.7 ± 20.8	17.3 ± 13.0	7.8 ± 13.0	2.8 ± 6.2	2.0 ± 5.6	<0.001	16.1 ± 19.6
Nuts and seeds	8.3 ± 15.0	12.4 ± 22.4	8.2 ± 16.0	6.9 ± 14.4	8.0 ± 13.6	<0.001	8.6 ± 16.6	6.8 ± 11.7	7.1 ± 9.8	6.1 ± 12.3	4.2 ± 10.1	4.3 ± 8.1	<0.001	7.8 ± 11.9
Soy products	17.7 ± 29.6	19.6 ± 33.1	9.2 ± 23.6	0.9 ± 6.0	0.5 ± 5.6	<0.001	18.2 ± 28.7	14.7 ± 34.6	21.4 ± 17.1	5.6 ± 17.2	0.7 ± 4.6	0.6 ± 5.3	<0.001	16.6 ± 50.4
Meat and fish intake														
Total fresh meat	0	0	16.4 ± 16.7	80.3 ± 32.2	119 ± 39	<0.001	10.4 ± 27.5	0	0	15.4 ± 15.1	73.5 ± 29.7	109 ± 36.1	<0.001	6.7 ± 18.0
Total fresh red meat	0	0	11.4 ± 12.6	68.9 ± 31.3	107 ± 39	<0.001	7.6 ± 23.6	0	0	9.7 ± 9.6	61.4 ± 28.8	96.0 ± 35.8	<0.001	4.4 ± 13.7
Beef	0	0	2.8 ± 4.6	20.8 ± 19.2	32.5 ± 26.3	<0.001	2.5 ± 9.9	0	0	2.8 ± 5.3	18.8 ± 17.3	29.4 ± 25.1	<0.001	1.5 ± 5.8
Pork	0	0	3.9 ± 5.8	27.9 ± 22.7	47.0 ± 30.6	<0.001	2.5 ± 10.1	0	0	2.9 ± 4.6	24.8 ± 20.5	43.0 ± 29.8	<0.001	1.3 ± 6.3
Minced meat	0	0	3.11 ± 5.6	15.7 ± 14.0	22.1 ± 18.9	<0.001	2.2 ± 7.5	0	0	2.8 ± 4.9	13.9 ± 12.7	19.3 ± 16.8	<0.001	1.2 ± 5.0
Liver	0	0	0.8 ± 3.7	1.9 ± 4.4	2.3 ± 4.8	<0.001	0.1 ± 0.6	0	0	0.5 ± 1.8	1.7 ± 3.8	1.9 ± 4.2	<0.001	0.2 ± 1.2
Chicken	0	0	5.6 ± 10.2	12.6 ± 14.3	13.9 ± 15.4	<0.001	2.8 ± 9.6	0	0	6.0 ± 11.6	12.9 ± 14.9	14.1 ± 16.5	<0.001	2.5 ± 8.6
Processed meat	0	0	4.6 ± 8.8	12.7 ± 14.6	18.1 ± 17.7	<0.001	1.9 ± 7.5	0	0	3.3 ± 8.3	8.4 ± 9.5	11.8 ± 12.4	<0.001	0.9 ± 4.5
Fish	0	32.0 ± 42.5	18.4 ± 27.2	17.0 ± 20.3	13.0 ± 15.0	<0.001	11.6 ± 23.3	0	23.9 ± 27.1	14.7 ± 23.5	13.8 ± 16.4	10.8 ± 13.1	<0.001	8.6 ± 16.6
Dairy products	356 ± 225	342 ± 244	335 ± 250	304 ± 203	306 ± 212	<0.001	347 ± 232	358 ± 220	341 ± 228	335 ± 233	311 ± 198	295 ± 186	<0.001	356 ± 220
Fermented milk products	151 ± 151	136 ± 149	125 ± 141	90.5 ± 111	81.7 ± 113	<0.001	144 ± 148	195 ± 167	190 ± 176	157 ± 158	124 ± 131	118 ± 126	<0.001	194 ± 163
Nonfermented milk products	205 ± 189	205 ± 191	210 ± 216	214 ± 175	225 ± 191	<0.001	203 ± 193	162 ± 171	151 ± 161	177 ± 182	186 ± 157	177 ± 152	<0.001	161 ± 162
Cheese	42.8 ± 34.9	45.1 ± 34.0	35.9 ± 33.2	24.8 ± 21.0	23.1 ± 20.5	<0.001	40.5 ± 33.2	38.5 ± 24.1	36.0 ± 24.4	31.9 ± 26.2	22.5 ± 17.7	21.5 ± 17.3	<0.001	37.0 ± 26.1
Eggs	14.4 ± 14.1	16.3 ± 13.6	14.0 ± 12.6	16.5 ± 11.1	17.4 ± 13.0	<0.001	15.0 ± 12.8	13.1 ± 13.1	14.0 ± 11.3	14.0 ± 12.9	14.8 ± 9.2	14.7 ± 10.1	<0.001	14.4 ± 11.9
Alcohol	4.3 ± 8.3	11.7 ± 18.5	8.6 ± 13.3	12.4 ± 14.4	16.1 ± 17.4	<0.001	6.8 ± 11.0	2.0 ± 5.5	4.5 ± 8.9	3.7 ± 8.3	5.6 ± 9.1	6.2 ± 10.2	<0.001	3.1 ± 7.0
Nutrients (energy percent)														
Fat total	40.7 ± 12.9	38.6 ± 13.4	44. 6 ± 17.9	43.3 ± 12.6	40.6 ± 11.0	<0.001	40.5 ± 12.5	41.8 ± 17.2	40.4 ± 16.3	43.7 ± 17.5	42.2 ± 12.5	41.3 ± 11.3	<0.001	40.2 ± 14.3
Mono unstaturated fat	13.5 ± 5.1	13.0 ± 5.2	15.6 ± 6.9	16.0 ± 5.1	15.5 ± 4.5	<0.001	13.7 ± 4.9	13.8 ± 6.6	13.5 ± 6.2	15.0 ± 6.6	15.5 ± 5.0	15.6 ± 4.6	<0.001	13.4 ± 5.4
Polyunsaturated fat	9.3 ± 4.4	9.5 ± 4.0	10.0 ± 5.2	9.5 ± 4.1	8.2 ± 3.7	<0.001	9.2 ± 4.0	8.9 ± 4.8	9.1 ± 4.8	9.6 ± 5.3	8.8 ± 4.2	8.1 ± 3.6	<0.001	8.7 ± 4.3
Saturated fat	16.3 ± 5.8	15.5 ± 6.0	17.7 ± 7.0	16.8 ± 5.2	15.9 ± 4.7	<0.001	16.1 ± 5.6	17.4 ± 7.2	16.3 ± 6.6	17.9 ± 7.4	17.0 ± 5.3	16.6 ± 4.9	<0.001	16.5 ± 6.2
Protein total	15.4 ± 4.2	15.9 ± 5.2	16.2 ± 5.7	15.4 ± 4.6	14.7 ± 4.2	<0.001	15.8 ± 4.4	16.4 ± 5.1	17.4 ± 5.8	16.5 ± 5.6	16.3 ± 4.9	16.4 ± 4.7	0.05	16.4 ± 4.7
Plant protein	8.7 ± 2.6	8.3 ± 3.2	7.8 ± 3.0	6.0 ± 1.8	5.2 ± 1.6	<0.001	8.4 ± 2.8	8.2 ± 2.8	8.5 ± 3.9	7.3 ± 2.8	5.8 ± 1.8	5.3 ± 1.6	<0.001	8.0 ± 2.7
Animal protein	6.8 ± 3.2	7.6 ± 3.8	8.5 ± 3.9	9.5 ± 3.4	9.7 ± 3.2	<0.001	7.4 ± 3.5	8.2 ± 3.9	9.0 ± 3.9	9.3 ± 4.0	10.6 ± 3.7	11.3 ± 3.7	<0.001	8.5 ± 3.7
Plant:animal ratio	42.0 ± 584	1.5 ± 1.5	1.1 ± 0.8	0.7 ± 0.3	0.6 ± 0.2	<0.001	22.5 ± 421	1.4 ± 4.1	0.8 ± 10.2	0.9 ± 1.3	0.6 ± 0.6	0.5 ± 0.2	<0.001	1.2 ± 2.7
Carbohydrates total	57.8 ± 15.7	52.5 ± 16.0	57.7 ± 20.7	48.9 ± 14.0	42.0 ± 12.0	<0.001	55.0 ± 15.4	56.0 ± 17.3	55.0 ± 18.1	54.9 ± 19.2	47.3 ± 12.6	42.7 ± 11.6	<0.001	53.0 ± 15.3
Mono- disacharides	27.8 ± 10.4	22.4 ± 9.0	25.9 ± 12.0	22.6 ± 8.6	19.4 ± 7.8	<0.001	23.7 ± 9.4	25.9 ± 10.3	24.9 ± 10.7	25.8 ± 11.2	22.6 ± 7.7	20.3 ± 7.4	<0.001	24.5 ± 9.4
Polysacharides	31.9 ± 8.6	28.7 ± 9.90	31.2 ± 11.6	26.1 ± 8.1	22.5 ± 6.9	<0.001	30.1 ± 8.8	29.0 ± 9.8	28.9 ± 9.9	28.7 ± 10.9	24.7 ± 7.6	22.4 ± 6.7	<0.001	27.5 ± 8.5

Values are means ± SD.

^a^Excluding prevalent cancer cases at baseline.

^b^P values are derived from one way anova comparing the five meat consumption groups (vegetarian, pescetarian, 1 day/wk-, 2–5 day/wk-, and 6–7 day/wk meat consumers).

**Table 5:** **Mean daily intake of selected vitamins, minerals, supplement use and salt intake of members of the NLCS-Meat Investigation Cohort by meat consumption status**^**a**^

	**Men**							**Women**						
	**All confirmed vegetarians, pescetarians and low meat consumers from the total cohort**			**Random sample of meat consumers from the total cohort**			**Self-reported vegetarians**	**All confirmed vegetarians, pescetarians and low meat consumers from the total cohort**			**Random sample of meat consumers from the total cohort**			**Self-reported vegetarians**
	**Vegetarian**	**Pescetarian**	**1 day/wk**	**2–5 day/wk**	**6–7 day/wk**			**Vegetarian**	**Pescetarian**	**1 day/wk**	**2–5 day/wk**	**6–7 day/wk**		
	**n = 217**	**n = 152**	**n = 482**	**n = 1326**	**n = 2973**	***P***^***b***^	**n = 416**	**n = 485**	**n = 242**	**n = 914**	**n = 1639**	**n = 2676**	***P***^***b***^	**n = 734**
Vitamins (from diet)														
Retinol (mg)	1.14 ± 0.35	1.11 ± 0.37	1.09 ± 0.38	1.05 ± 0.37	1.01 ± 0.37	<0.001	1.14 ± 0.34	1.04 ± 0.36	1.05 ± 0.39	0.99 ± 0.37	0.93 ± 0.37	0.90 ± 0.38	<0.001	1.07 ± 0.37
β-carotene (mg)	0.61 ± 0.35	0.59 ± 0.37	0.49 ± 0.32	0.42 ± 0.23	0.40 ± 0.21	<0.001	0.60 ± 0.34	0.60 ± 0.36	0.64 ± 0.39	0.52 ± 0.34	0.44 ± 0.26	0.41 ± 0.23	<0.001	0.64 ± 0.37
Vitamin B1 (mg)	1.25 ± 0.26	1.23 ± 0.25	1.14 ± 0.23	1.15 ± 0.19	1.22 ± 0.22	<0.001	1.25 ± 0.25	1.04 ± 0.24	1.03 ± 0.21	0.96 ± 0.20	0.99 ± 0.17	1.06 ± 0.19	<0.001	1.04 ± 0.22
Vitamin B2 (mg)	1.62 ± 0.38	1.63 ± 0.39	1.61 ± 0.42	1.59 ± 0.37	1.57 ± 0.36	<0.001	1.63 ± 0.39	1.51 ± 0.39	1.52 ± 0.38	1.48 ± 0.41	1.47 ± 0.36	1.44 ± 0.34	<0.001	1.52 ± 0.38
Vitamin B3 (mg)	14.7 ± 3.6	16.7 ± 5.0	16.2 ± 4.4	15.6 ± 3.8	15.4 ± 4.1	<0.001	15.4 ± 3.8	11.8 ± 3.2	12.9 ± 3.1	12.6 ± 3.3	12.6 ± 2.9	12.4 ± 3.0	<0.001	12.2 ± 3.2
Vitamin B6 (mg)	1.42 ± 0.27	1.40 ± 0.29	1.37 ± 0.31	1.48 ± 0.25	1.57 ± 0.28	<0.001	1.45 ± 0.29	1.15 ± 0.25	1.17 ± 0.23	1.17 ± 0.27	1.28 ± 0.23	1.37 ± 0.24	<0.001	1.19 ± 0.26
Vitamin B11 (μm)	252 ± 55	250 ± 61	232 ± 69	227 ± 64	222 ± 65	<0.001	253 ± 57	217 ± 60	218 ± 52	204 ± 62	200 ± 59	197 ± 62	<0.001	223 ± 58
Vitamin C (mg)	123 ± 51	122 ± 57	108 ± 51	101 ± 42	97 ± 41	<0.001	124 ± 52	127 ± 54	122 ± 50	117 ± 56	109 ± 44	106 ± 42	<0.001	128 ± 51
Vitamin E (mg)	16.1 ± 6.0	15.3 ± 5.1	15.4 ± 5.1	15.4 ± 5.6	14.4 ± 5.6	<0.001	16.2 ± 5.4	12.9 ± 4.4	13.1 ± 4.4	12.9 ± 4.9	12.4 ± 4.6	11.8 ± 4.4	<0.001	13.2 ± 4.4
Minerals														
Calcium (mg)	1238 ± 379	1242 ± 395	1160 ± 380	991 ± 291	934 ± 294	<0.001	1211 ± 391	1144 ± 327	1117 ± 324	1062 ± 335	927 ± 275	875 ± 263	<0.001	1125 ± 323
Phosphor (mg)	1726 ± 293	1758 ± 312	1647 ± 313	1542 ± 256	1528 ± 255	<0.001	1728 ± 309	1453 ± 260	1471 ± 250	1388 ± 282	1324 ± 240	1318 ± 228	<0.001	1460 ± 257
Magnesium (mg)	435 ± 103	449 ± 120	389 ± 91	355 ± 59	329 ± 53	<0.001	436 ± 104	360 ± 87	369 ± 87	322 ± 72	292 ± 47	289 ± 46	<0.001	363 ± 85
Iron (mg)	14.4 ± 2.8	15.0 ± 2.9	13.6 ± 2.7	13.2 ± 2.3	13.3 ± 2.4	<0.001	14.6 ± 2.6	11.8 ± 2.3	12.3 ± 2.4	11.4 ± 2.1	11.5 ± 2.0	11.7 ± 2.0	<0.001	12.0 ± 2.2
Potassium (mg)	3768 ± 634	3771 ± 749	3656 ± 727	3684 ± 585	3763 ± 605	<0.001	3796 ± 673	3261 ± 607	3312 ± 618	3229 ± 658	3288 ± 532	3348 ± 544	<0.001	3313 ± 598
Dietary cholesterol (mg)	197 ± 83	228 ± 84	227 ± 73	265 ± 67	287 ± 78	<0.001	213 ± 81	177 ± 66	187 ± 66	200 ± 67	229 ± 58	245 ± 61	<0.001	188 ± 66
Dietary fiber (g)	40.3 ± 8.8	39.3 ± 9.6	34.8 ± 9.0	29.8 ± 7.3	28.1 ± 6.8	<0.001	39.6 ± 8.9	32.6 ± 7.3	32.5 ± 7.0	29.3 ± 7.1	25.6 ± 5.8	24.6 ± 5.5	<0.001	32.4 ± 7.1
Supplement use (number of sup/day)	0.9 ± 1.2	0.9 ± 1.2	0.8 ± 1.1	0.4 ± 0.9	0.3 ± 0.6	<0.001	0.9 ± 1.2	1.3 ± 1.3	1.3 ± 1.3	1.0 ± 1.3	0.6 ± 0.9	0.5 ± 0.8	<0.001	1.33 ± 1.3
NaCl (mg) (from foods)	5800 ± 2150	6496 ± 2331	6127 ± 2410	6316 ± 2054	6693 ± 2231	<0.001	6073 ± 2302	4678 ± 1743	4964 ± 1740	5203 ± 9097	5158 ± 1639	5433 ± 1813	<0.001	4869 ± 1672
Salt added during cooking (g)	1.67 ± 2.58	1.96 ± 2.07	2.65 ± 3.22	4.01 ± 4.06	4.43 ± 4.32	<0.001	1.94 ± 2.69	1.55 ± 2.34	2.14 ± 2.51	3.00 ± 4.13	3.86 ± 4.05	3.99 ± 4.26	<0.001	1.94 ± 2.69

Values are mean ± SD.

Nutrients are adjusted for total energy intake (using residuals).

^a^Excluding prevalent cancer cases at baseline.

^b^P values are derived from one way anova comparing the five meat consumption groups (vegetarian, pescetarian, 1 day/wk-, 2–5 day/wk-, and 6–7 day/wk meat consumers).

### Time on diet

The time of adherence to diet was significantly different between vegetarians, pescetarians and those consuming meat 1 day/week (P < 0.001) (Table 6). Both vegetarian men and women were more often on a dietary regimen for a longer period of time (>15 yrs) than pescetarians and low meat consumers.

**Table 6:** **Time on diet by meat consumption status**^**a**^

	**Men**					**Women**				
	**All confirmed vegetarians, pescetarians and low meat consumers from the total cohort**				**Self-reported vegetarians**	**All confirmed vegetarians, pescetarians and low meat consumers from the total cohort**				**Self-reported vegetarians**

	**Vegetarian**	**Pescetarian**	**1 day/wk**			**Vegetarian**	**Pescetarian**	**1 day/wk**		
	**n = 217**	**n = 152**	**n = 482**	***P***^***b***^	**n = 416**	**n = 485**	**n = 242**	**n = 914**	***P***^***b***^	**n = 734**
Time of adherence to special eating habit (%)										
≤ 5 yrs	9%^c^	28%	27%	<0.001	18%	16%	33%	27%	<0.001	21%
6–10 yrs	23%	27%	22%		23%	25%	27%	26%		25%
11–15 yrs	18%	13%	14%		16%	16%	16%	14%		16%
≥16 yrs	44%	25%	23%		34%	37%	17%	19%		30%
No data available	7%	7%	14%		9%	6%	7%	14%		9%

^a^Excluding prevalent cancer cases at baseline.

^b^P values are derived from a chi2 tests comparing vegetarians, pescetarians, 1 day/wk meat consumers.

^c^% of total vegetarians, all such values.

### Prevalent cancer cases (results not shown)

The proportion of prevalent cancer cases was highest among the vegetarian and pescetarians (both 11%) and significantly decreased with increasing meat intake (1 day/week (7%), 2–5 days/week (5%), and 6-7 days/week (4%) (P < 0.001) (Additional file 1: Figure S1)). Vegetarians who had been diagnosed with cancer before baseline (prevalent cancer cases) were more often female and showed a more favorable distribution of lifestyle factors compared to vegetarians without a cancer diagnosis; they were more physically active and less likely to be current smokers. As could be expected, prevalent cancer cases scored their general health status considerably lower, and spent more hours per day sleeping. Vegetarians with a prevalent cancer were more extreme in their dietary intakes compared to those without a cancer diagnosis; they ate substantially more fruits, vegetables, pulses, grains, nuts and seeds, dairy cheese, eggs, proteins and carbohydrates, but less fat and alcohol. Moreover, 65% of vegetarians with a prevalent cancer reported using a nutritional supplement compared to 58% in the cancer free vegetarians. Three quarters of the vegetarians and 1 day/week meat consumers with a cancer diagnosis at baseline had started their dietary regimen in the same year or the year following their cancer diagnosis. This was also reflected in their shorter time of adherence to their special eating habits compared to cancer free individuals.

### Cross-comparison of self-reported vegetarian status and FFQ data

We observed that 50% of all self-reported vegetarians reported meat consumption on the FFQ. However, self-reported vegetarians who reported meat of fish consumption on the FFQ did not differ much with respect to lifestyle and dietary characteristics compared to self-reported vegetarians who abstained from all meat and fish products. Moreover, the self-reported vegetarians who reported meat consumption on the FFQ, ate very little meat at rates comparable to the 1 day/week meat consumers (results not shown). Among women, compared to self-reported vegetarians who reported meat or fish consumption on the FFQ, confirmed vegetarians were significantly less often current smokers (15% and 6%, respectively (P < 0.001), results not shown). Although confirmed self-reported vegetarians had a higher level of education (P < 0.001) compared to the self-reported vegetarians who reported meat or fish consumption on the FFQ, this latter group still had a higher education level than any of the meat consumption groups (results not shown). Meat eating self-reported vegetarians had higher intakes of vitamin B3, B6, cholesterol and a lower intake of dietary fiber, and added more salt to their meal during cooking compared to confirmed self-reported vegetarians (P < 0.001) (results not shown).

## Discussion

We defined an analytical cohort with a wide range of dietary intake by over-representing our study population with persons at the lower end of the meat consumption spectrum. The dietary intake patterns and lifestyle characteristics of vegetarians, pescetarians and those consuming meat 1 day/week were diverse and distinct from individuals consuming meat on a regular basis. A cross-comparison between self-reported vegetarians and vegetarians whose status was confirmed based on extensive FFQ data, did not show large differences between both groups in terms of diet and lifestyle characteristics.

To date, no universally accepted definition for the term vegetarian exists and the only constant component in a vegetarian diet across all previous empirical studies has been the absence of meat. As previously outlined by Fraser, the problem of defining a dietary regimen on just one food group results in a lack of control on intake of all other food groups that make up a vegetarian diet [24]. Consequently, all individuals who refrain from meat, but have otherwise quite distinct dietary intakes, are grouped together under one label of vegetarianism. We included a large number of individuals consuming meat only once a week with the purpose to address whether low meat consumption might have a beneficial health effect over complete abstinence of meat, or vice versa. Despite an increase in the number of low meat consumers and meat reducers in the Western world [25], little to no research has been conducted in this area. However, we showed no large differences in terms of nutrient intake between these groups, though we had no data on vitamin B12 intake [26].

Interestingly, we showed that the perceived general health of vegetarians is considerably better than that of non-vegetarians. This is in line with our finding that vegetarianism is not merely characterized by a diet void of all flesh foods, but rather extends into a complete healthy lifestyle. In our population, we found evidence for a higher level of health consciousness among the vegetarians, pescetarians and to a lesser extent low meat consumers, as indicated by a more favorable distribution of dietary and lifestyle factors. Although the diet groups were statistical significantly different with respect to most of the dietary variables, this may have resulted from our large sample size. Interestingly, the amount of salt added to the food during cooking increased with increasing animal product intake in a dose–response relation. Pescetarians had higher intakes of soy products, nuts and seeds, grains, cheese and eggs than vegetarians, which would suggest that they more actively seek to replace the meat in the diet with plant based protein-rich foods to balance their diets.

Appropriately planned vegetarian diets have shown to be consistent with the current dietary guidelines in all stages of the lifecycle [27]. The mean nutrient intakes (not from supplements) in all diet groups were generally well above the recommended daily allowances (RDA) of the European Commission [28], except for iron for which the intakes were below the RDA of 14 mg/day in the meat eating men and all women. The estimated iron intake was highest among pescetarians and vegetarians. However, these groups consume predominantly inorganic iron which has a low bioavailability compared to the heme-bound iron that is found in meat products [29]. More than 55% of the vegetarians and pescetarians reported to take a nutritional supplement over the last year compared to 26% of individuals in the highest meat consumption group (6-7 days/week). This suggests that there appears to be a certain degree of awareness among these individuals of the need to supplement their diets to prevent dietary deficiencies.

Although literature suggests that long-term adherence to a vegetarian diet appears more strongly associated with health outcomes than short term adherence [11,30], very few prospective studies in vegetarian populations have specific data available on time since adopting the diet. On average, vegetarians adhered to their diet considerably longer than pescetarians and individuals consuming meat 1 day/week. As much as 75% of all confirmed-vegetarians who had been diagnosed with cancer before baseline changed to this dietary regimen after diagnosis. In addition, the proportion of prevalent cancer cases decreased with increasing meat intake. Previous research indicates that cancer survivors are highly motivated to make dietary changes towards a more plant-based diet after diagnosis with the intention to improve their health and well-being [31]. However, although nutrition has shown to affect cancer progression [32], it remains to be elucidated whether adopting a vegetarian lifestyle may influence the course of cancer prognosis or cancer recurrence.

Nearly half of those who called themselves vegetarian reported to consume fish, meat or poultry on the extensive FFQ (ĸ = 0.59). Similar findings have been reported previously [19,20] and indicate that the complete avoidance of meat cannot be assumed among self-reported vegetarians. This suggests that self-identification is not a good measure for estimating the prevalence of vegetarianism. However, our findings suggest that the level of misclassification that occurs when merely relying on self-reported vegetarian status was small: the overall group of self-reported vegetarians did not differ considerably from individuals whose vegetarian status has been confirmed based on FFQ data in terms of diet and lifestyle. The FFQ assessed the diet in the past 12 months whereas self-definition was based on vegetarian status at the time of the questionnaire. Nonetheless, only 10% of the observed disagreement could be explained by this difference in time-frame. It would be interesting to examine the difference in risk ratios between both groups of vegetarians in future etiological studies of chronic diseases. After 20.3 yrs of follow-up, a total of 1,559 incident cancer cases (165 among vegetarians and 346 among 1 day/week meat consumers) were identified in NLCS-MIC through record linkage with the Netherlands Cancer Registry.

An important methodological issue when comparing previous studies on the health effects of vegetarian diets relates to between-study sampling differences. Only a few reports on vegetarian diets, including ours, are from population-based studies while the majority stem from convenience samples that have likely also recruited more health-conscious non-vegetarians [5-8,10,33]. The latter technique is particularly appropriate to recruit non vegetarians who only differ from vegetarians with respect to their meat and fish intake and is a suitable design for studies into diet and health in vegetarian populations that are mainly concerned with the adverse effects of meat. However, interest has shifted towards the health effects of the complete vegetarian lifestyle. For this, convenience sampling may be less appropriate since it likely decreases diet and lifestyle differences between vegetarians and non-vegetarians, and could bias results towards the null. Our population has a wide distribution of nutrient intakes and lifestyle characteristics, which should facilitate the identification of associations between vegetarianism, meat consumption and disease risk in future etiologic studies. The ratio of low meat consumers to high meat consumers (meat consumption ≤1 day/week versus 6–7 days/week) was 1:23 in the total NLCS cohort and 1:2.2 in NLCS-MIC.

The NLCS aimed to overrepresent vegetarians by intentionally contacting them through health food shops and magazines. Therefore, vegetarian dietary patterns were taken into consideration when designing the FFQ by including line items on meat substitutes that were commonly used by the vegetarian population at that time. Vegetarians and low meat consumers more often took the opportunity to report and give details of foods and beverages that were frequently eaten but that were not contained in the FFQ. Moreover, vegetarian status was taken into account for nutrient calculation of composite recipes. Previous studies indicate that vegetarians are able to recall their diet with higher reliability [34] but at the same time may be more tempted to report the intake of certain food items that they consider to be healthy as a result of social desirability bias [20]. The FFQ used in the NLCS was not designed for assessing the usefulness of self-reported vegetarianism as a classification tool. Interestingly, some 18% of individuals who were classified as vegetarians based on their responses on the FFQ did not report to have any special eating habits. This phenomenon has previously been reported [20] and may result from lack of knowledge of the concept of vegetarianism by the general public at the time the measurement was conducted (1986).

## Conclusion

With NLCS-MIC we successfully established a cohort comprising a considerable number of vegetarians, pescetarians, and 1 day/week meat consumers. The wide distribution of dietary and lifestyle characteristics within the cohort should facilitate the identification of associations between vegetarianism, meat consumption and the risk of major types of cancer and cause-specific mortality using ≥20.3 yrs of follow-up in future etiologic studies.

## Abbreviations

ANOVA: Analysis of variance; BMI: Body mass index; FFQ: Food frequency questionnaire; NLCS: Netherlands cohort study; NLCS-MIC: Netherlands cohort study – Meat investigation cohort; RDA: Recommended daily allowance.

## Competing interests

The authors declare that they have no competing interests.

## Authors’ contributions

The authors’ responsibilities were as follows - AMJG carried out the statistical analysis, interpreted the data and drafted the manuscript. MPW, LJS, and PCD assisted with the data interpretation and critically revised the manuscript. PAvdB and RAG conceived the study, participated in the design and coordination of the study and critically revised the manuscript. All authors approved the final version of the manuscript and none of the authors had a conflict of interest.

## Supplementary Material

Additional file 1: Figure S1 Flowchart describing the classification of the meat consumption categories in the NLCS-Meat Investigation Cohort. For detailed legend see web appendix 1. Click here for file
