# Supplementary material for: The Netherlands Cohort Study – Meat Investigation Cohort; a population-based cohort over-represented with vegetarians, pescetarians and low meat consumers
Source: Nutr J. 2013 Nov 29;12:156. doi: 10.1186/1475-2891-12-156 (PMC4220685; doi:10.1186/1475-2891-12-156)
Supplement: Additional file 1: Figure S1 — Flowchart describing the classification of the meat consumption categories in the NLCS-Meat Investigation Cohort. For detailed legend see web appendix 1. [file 1475-2891-12-156-S1.pdf]

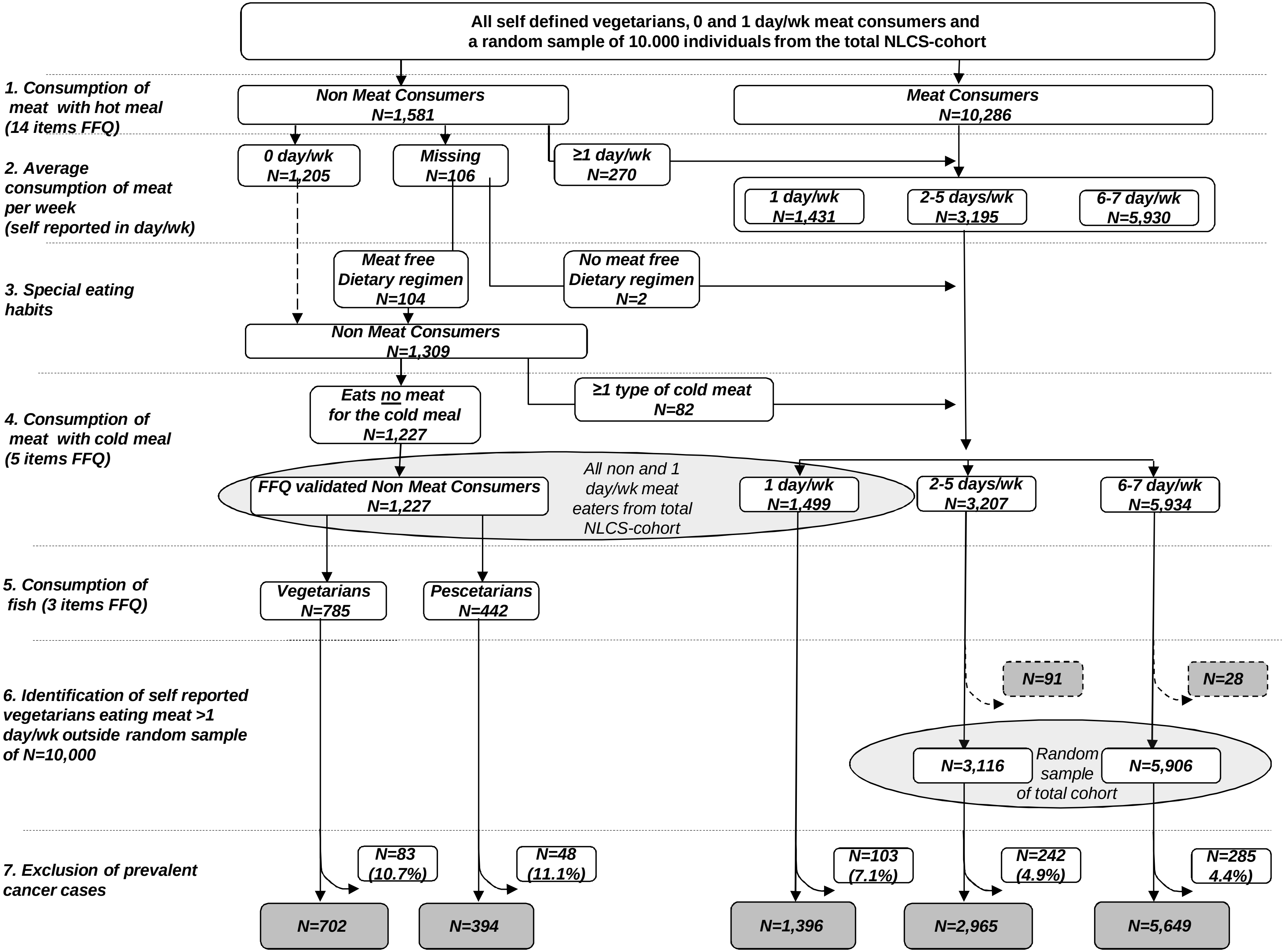

Supplemental Figure 1: flowchart describing the classification of the meat consumption categories in the NLCS-Meat Investigation Cohort. For detailed legend see web appendix 1.
